# Fractal-based analysis of optical coherence tomography data to quantify retinal tissue damage

**DOI:** 10.1186/1471-2105-15-295

**Published:** 2014-09-01

**Authors:** Gábor Márk Somfai, Erika Tátrai, Lenke Laurik, Boglárka E Varga, Vera Ölvedy, William E Smiddy, Robert Tchitnga, Anikó Somogyi, Delia Cabrera DeBuc

**Affiliations:** Department of Ophthalmology, Faculty of Medicine Semmelweis University, Budapest, Hungary; Miller School of Medicine, Bascom Palmer Eye Institute, University of Miami, Miami, Florida 33136 USA; Faculty of Science, Department of Physics, University of Dschang, Dschang, Cameroon; 2nd Department of Internal Medicine, Faculty of Medicine Semmelweis University, Budapest, Hungary

**Keywords:** Optical coherence tomography, Fractal analysis, Fractal dimension, Wavelet algorithm, Diabetic retinopathy, Ophthalmology

## Abstract

**Background:**

The sensitivity of Optical Coherence Tomography (OCT) images to identify retinal tissue morphology characterized by early neural loss from normal healthy eyes is tested by calculating structural information and fractal dimension. OCT data from 74 healthy eyes and 43 eyes with type 1 diabetes mellitus with mild diabetic retinopathy (MDR) on biomicroscopy was analyzed using a custom-built algorithm (OCTRIMA) to measure locally the intraretinal layer thickness. A power spectrum method was used to calculate the fractal dimension in intraretinal regions of interest identified in the images. ANOVA followed by Newman-Keuls post-hoc analyses were used to test for differences between pathological and normal groups. A modified p value of <0.001 was considered statistically significant. Receiver operating characteristic (ROC) curves were constructed to describe the ability of each parameter to discriminate between eyes of pathological patients and normal healthy eyes.

**Results:**

Fractal dimension was higher for all the layers (except the GCL + IPL and INL) in MDR eyes compared to normal healthy eyes. When comparing MDR with normal healthy eyes, the highest AUROC values estimated for the fractal dimension were observed for GCL + IPL and INL. The maximum discrimination value for fractal dimension of 0.96 (standard error =0.025) for the GCL + IPL complex was obtained at a FD ≤ 1.66 (cut off point, asymptotic 95% Confidence Interval: lower-upper bound = 0.905-1.002). Moreover, the highest AUROC values estimated for the thickness measurements were observed for the OPL, GCL + IPL and OS. Particularly, when comparing MDR eyes with control healthy eyes, we found that the fractal dimension of the GCL + IPL complex was significantly better at diagnosing early DR, compared to the standard thickness measurement.

**Conclusions:**

Our results suggest that the GCL + IPL complex, OPL and OS are more susceptible to initial damage when comparing MDR with control healthy eyes. Fractal analysis provided a better sensitivity, offering a potential diagnostic predictor for detecting early neurodegeneration in the retina.

## Background

Optical coherence tomography (OCT) is a real-time, noninvasive imaging modality that employs interferometry to detect backscattered near-infrared light to render two-dimensional (2D) or three-dimensional (3D) images of tissue. OCT is a powerful tool for retinal measurement [1]. Particularly, OCT has been used to measure volume and total thickness of the retina along with structural changes of the various cellular layers of the retina with the aid of segmentation algorithms [2, 3]. The role of OCT in the assessment and management of retinal diseases has become significant in understanding the vitreoretinal relationships and the internal architecture of the retinal structure. Particularly, structural information extracted from OCT retinal images has been used to characterize early neural loss in patients with diabetes and multiple sclerosis [4, 5]. The most important retinal pathology caused by diabetes is diabetic retinopathy (DR), which is characterized by blood vessels damage.

OCT has also improved diagnosis and management of retinal diseases by reducing reliance on insensitive tests such as perimetry and subjective disc grading. Though thickness differences may characterize regions with early pathological signs from normal regions, differences in optical properties and texture descriptors of normal and abnormal retinal tissue may also provide additional information of disease development in pathological eyes. The appropriateness of texture to classify tissues in OCT images has been shown in previous studies [6]. We have also shown that diabetic retinopathy not only causes thinning of the inner retinal layers, but also reduces the amplitude of the back-reflected signal from these layers [7–9]. Therefore, predictors based on optical properties changes are also of interest. Differences in optical properties and roughness measures of normal and abnormal retinal tissue may provide additional information of disease development in pathological eyes.

The fractal analysis of biological structures has been a continuous area under discussion ever since Mandelbrot’s famous essay [10]. Fractal analysis techniques are common tools in physics and image processing. Fractals are objects that show self-similarity at different magnifications. One of the advantages of fractal analysis is the ability to quantify the irregularity and complexity of objects with a measurable value, which is called the fractal dimension [10]. The fractal dimension is a measure of the roughness of a fractal structure. Higher values indicate rougher surface. Fractal dimension is regarded as local property of the system. Fractal analysis has also been used for the description of texture in medical images [11]. Texture can be defined as the spatial distribution of intensity values in an image. In our particular case, texture can be defined as the spatial distribution of intensity values in an OCT image, where the intensity at each pixel is the back-reflection of the incident light. The back-reflected light contains information of the retinal structure such as the directionality, function and dysfunction (in the case of pathological retina) of the cellular layers. In ophthalmology, a major interest has been focused on the fractal properties of the retinal vasculature especially for diagnosis purpose. Most of the studies have used differences in the fractal dimension as a discriminant factor to detect and diagnose eye disease [12–15]. In general, a global measure characterizing the whole branching pattern of the retinal vascular network has been used as a single parameter in these previous studies. However, the global analysis of the vascular network features may overlook the very early changes in the structure and, therefore, not be sensitive to the early manifestation of the particular disease. Up to now, fractal-based analysis of OCT data has been used to quantify photoreceptor rearrangement and vision restoration, identify early glaucomatous damage in the retinal nerve fiber layer and as an index for capillary integrity of pathological disorders [16–18]. However, it has not been implemented to differentiate normal healthy eyes from pathological eyes with early neural loss in multiple intraretinal layers (e.g. in DR and multiple sclerosis) using a local approach through segmentation of the various cellular layers of the retina and characterization of texture-based features on OCT intensity images.

In this study, the sensitivity of OCT images to identify retinal tissue morphology characterized by early neural loss in diabetes from normal healthy eyes is tested by calculating structural information and fractal dimension of the various cellular layers of the retina. Particularly, we found that fractal analysis provided a better sensitivity, offering a potential diagnostic predictor for detecting early neurodegeneration in the diabetic retina.

## Methods

In this study, we evaluated the diagnostic power of a novel method based on the fractal analysis of OCT-derived retinal tissue layer properties in discriminating normal healthy eyes from diabetic eyes with early neural loss. Although texture measures of the retinal tissue are not standardized measures for detecting significant intraretinal changes, texture-based measures were obtained from OCT intensity images and used in the fractal dimension analysis. In addition, the fractal analysis’ diagnostic outcome was compared with the standard approach that uses structural information extracted from OCT images. Specifically, we calculated fractal dimension and thickness using features measured locally for each intraretinal layer and evaluated their suitability to quantify retinal tissue damage.

### Study population

The study was approved by the Institutional Review Board in each institution involved in the study (University of Miami, Miami, FL, USA and Semmelweis University, Budapest, Hungary). The research adhered to the tenets set forth in the declaration of Helsinki and written informed consent was obtained from each subject. In this prospective study, enrollment was offered to type 1 diabetic patients referred to the comprehensive ophthalmology clinic that had diabetic retinopathy up to ETDRS level 35 and without macular edema, as well as type 1 diabetic patients with no retinopathy [19]. Patients with proliferative disease, clinically significant macular edema (CSME), and anatomic abnormalities that might confound evaluation of macular architecture, such as glaucoma, vitreoretinal traction and epiretinal membranes were excluded. Healthy controls were selected if best-corrected visual acuity was at least 20/25, a history of any current ocular or systematic disease was lacking, and the macula appeared normal when examined with contact lens biomicroscopy. Patients with medical conditions that might affect visual function, receiving treatments with medications that might affect retinal thickness (e.g. chloroquine or niacin containing anticholesterol agents), recent cataract surgery, previous vitrectomy, or unstable blood sugars were excluded.

Once enrolled a comprehensive eye examination was performed including slit lamp examination, measurement of intraocular pressure (using Goldmann tonometer), and fundus biomicroscopy. OCT imaging and 2 standard field stereoscopic fundus photos (SFPs) were obtained in all patients. The SFPs were classified by independent graders according to the criteria of proposed international clinical diabetic retinopathy and diabetic macular edema disease severity scales based on the ETDRS protocol [20, 21]. The graders were unaware of the OCT findings and clinical data. In addition, a hemoglobin A1c level test was required at this visit for diabetic patients.

### OCT data analysis and measurement of fractal dimension

The appropriateness of texture to classify tissues in OCT images has been shown in previous studies [6]. By analyzing the spatial arrangement of intensities in an image or selected region of interest (ROI), the image irregularities can be measured. Because the apparent reflectivity measured by OCT is a combination of the actual reflectivity and the scattering and absorption characteristics of the overlying media, the reflectivity measured by OCT may be affected by abnormalities in the retinal tissue. Consequently, structure disorder in the retinal tissue can be assessed when the fractal dimension is calculated using the intensity or reflectivity profile along the direction of depth in OCT images. Therefore, the fractal dimension was analyzed for each intraretinal layer segmented on OCT images and used as an indicator of retinal structure disorder or roughness measure.

**Figure 1:**
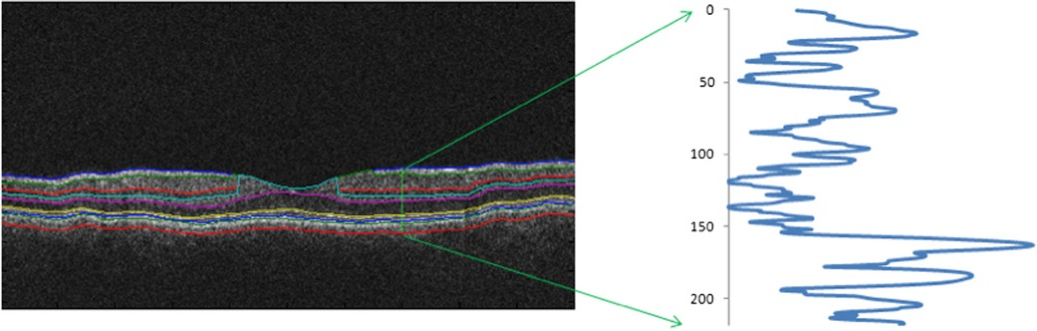
**Reflectivity profile used to calculate the fractal dimension.** The fractal dimension was calculated for the reflectivity profile within each intraretinal layer for each A-scan.

A method based on the power spectrum was used to calculate the fractal dimension in OCT images [22]. Since the average power spectrum of an image obeys a power law scaling, the fractal dimension was calculated from the power law detected in the graph of the power spectrum as a function of the frequency in the Fourier transform of the OCT image (gray scale). In this particular case, when the graph is plotted in a log-log scale the curve is approximately similar to a straight line and the dimension is provided by the slope of the line. The fast Fourier transform (FFT) was applied to the OCT reflectivity’s profiles (see Figure 1) to obtain the power spectrum as follows:

1

Where *P*(*ω*) is the power spectrum with the frequency *ω. β* is the spectral exponent of the reflectivity profile. The equation (1) can be converted into:
2

The fractal dimension is linked to the power-law exponent *β* by the following relationship [22]:
3

Therefore, the fractal dimension was evaluated from the slope *β* of a least-square regression line fit (polynomial regression of degree 1) to the data points in log-log plot of power spectrum. The fractal dimension was calculated for the reflectivity profile within each intraretinal layer for each A-scan (see Figure 1). The mean value of the fractal dimension was calculated by averaging the fractal dimension measurements across all A-scans in each macular region of each intraretinal layer. MATLAB software (The Mathworks, Natick, MA) was used to perform the fractal dimension analysis using a custom-built algorithm.

**Figure 2:**
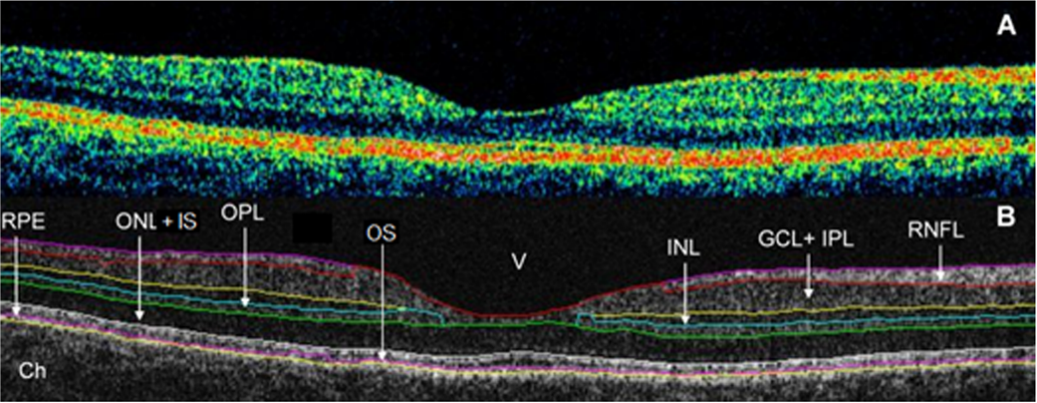
**Macular image segmentation results using OCTRIMA. (A)** The image of a healthy macula scanned by Stratus OCT. **(B)** The same OCT scan processed with OCTRIMA. Abbreviations: Ch, choroid; GCL + IPL, ganglion cell layer and inner plexiform layer complex; INL, inner nuclear layer; ONL + IS, combined outer nuclear layer and inner segment of photoreceptors; OS, outer segment of photoreceptors; OPL, outer plexiform layer; RNFL, retinal nerve fiber layer; RPE, retinal pigment epithelial layer; V, vitreous.

All Stratus OCT study cases were obtained using the “macular thickness” map protocol. This protocol consists of six radial scan lines centered on the fovea, each having a 6 mm transverse length. Macular radial line scans of the retina for each case were exported to disc with the export feature available in the Stratus OCT device and analyzed using a custom-built software (OCTRIMA) that facilitates the automatic segmentation of 7 cellular layers of the retina on OCT images based on their optical densities (see Figure 2). These retinal layers are the retinal nerve fiber layer (RNFL), the ganglion cell and inner plexiform layer complex (GCL + IPL), the inner nuclear layer (INL), the outer plexiform layer (OPL), the outer nuclear layer and inner photoreceptor segment (ONL + IS), outer photoreceptor segment (OS) and retinal pigment epithelium (RPE) [3]. Details of the methodology, such as segmentation, speckle noise removal and semiautomatic correction of discontinuities in each detected boundary after automated segmentation, along with manual error correction using direct visual evaluation of the detected boundaries, have been described in detail elsewhere [3–5, 7–9, 23–26].

Each OCT image used in this study was composed of 512 A-scans. Lateral coordinates of the blood vessel shadows were first extracted by using a blood vessel shadowgram technique and removed in each OCT image before calculating parameters related to reflectivity values [27].

**Figure 3:**
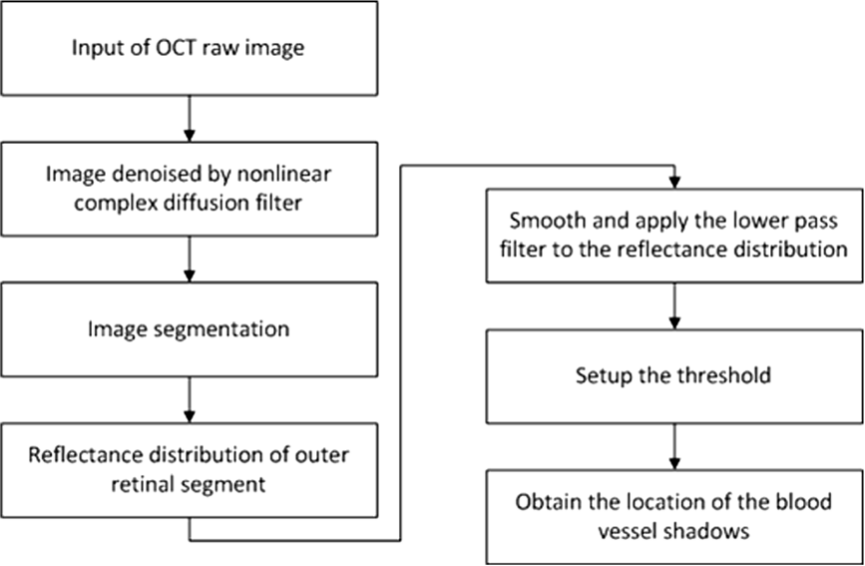
**Flowchart of the detection of blood vessel shadows in OCT images.**

**Figure 4:**
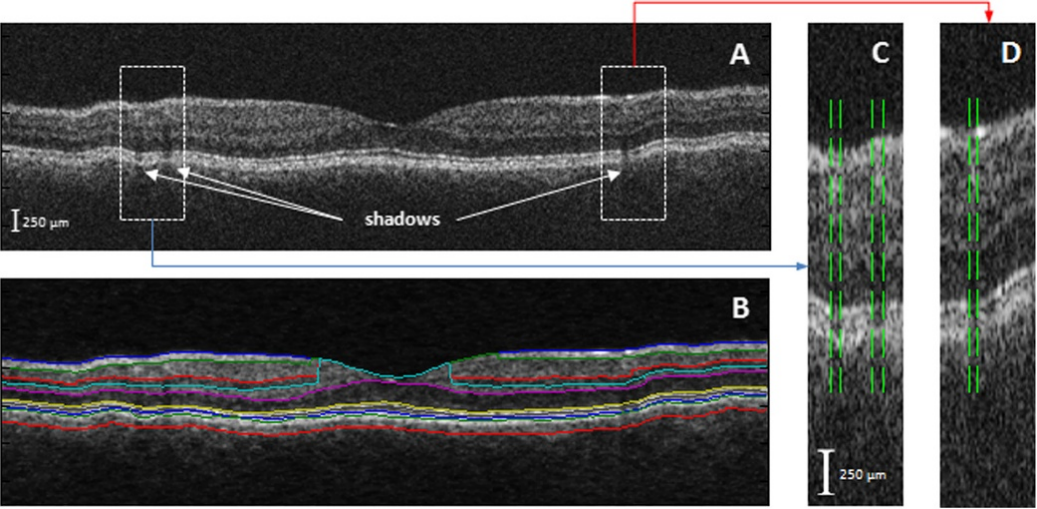
**An example of the detection of the blood vessel shadows by the shadowgram technique. A)** the raw OCT image of the macula. **B)** The same OCT image showing segmentation results after removal of speckle noise. **C-D)** Zoomed-in views of the shadowed regions are shown with the detected boundaries of blood vessel shadows.

In brief, we used the image gradient to detect edges such as the boundaries of blood vessel shadows for the shadowgram technique. With a proper threshold, locations of blood vessel shadows can be found in OCT images [27]. As the incident light perpendicularly penetrates into the retinal tissue, the direction of the blood vessel shadows’ boundaries are vertical in OCT images which was employed to detect the lateral coordinates of the blood vessel shadows [27]. The algorithm flowchart is shown on Figure 3 while Figure 4 shows an example of the use of the shadowgram technique.

**Figure 5:**
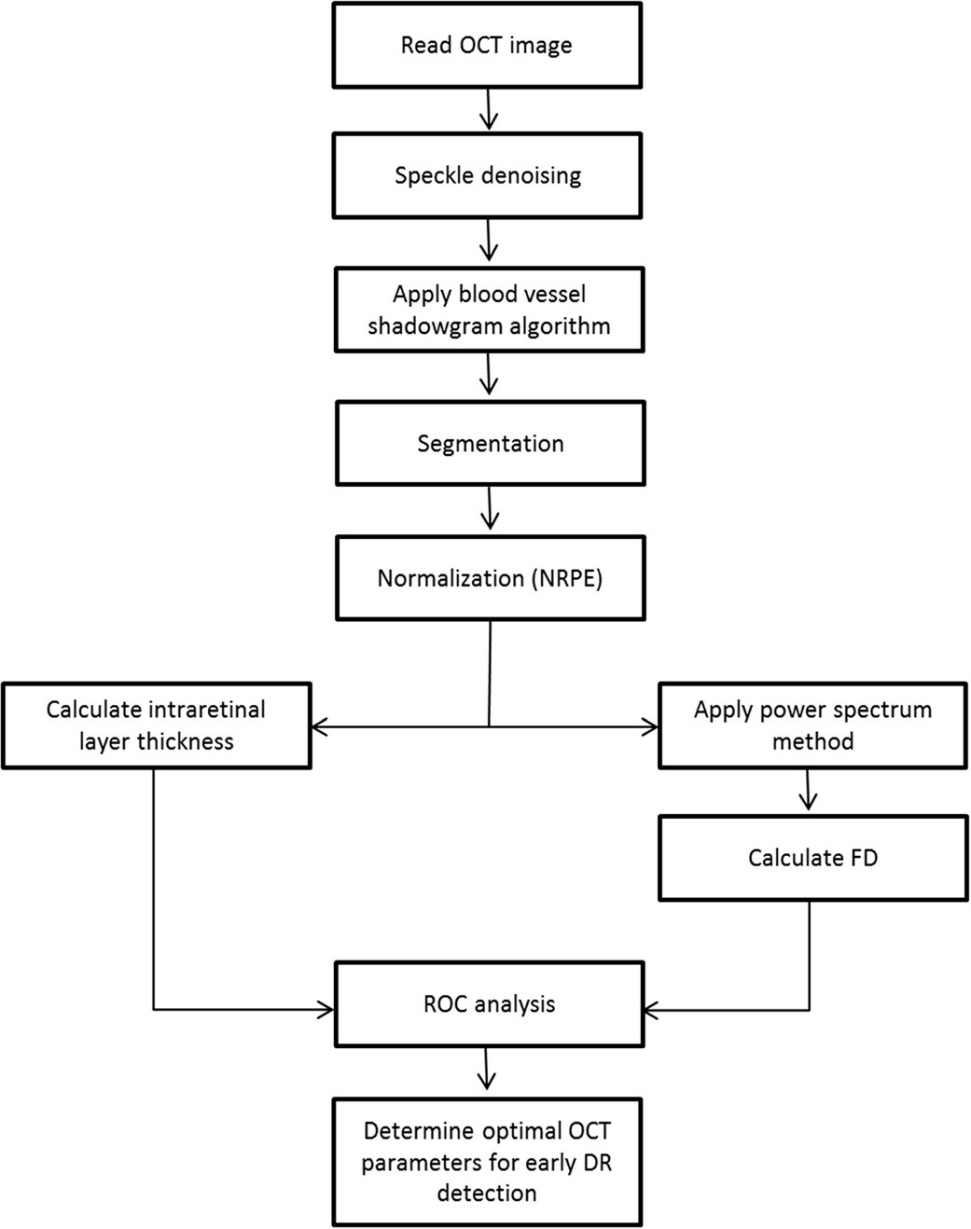
**Flowchart describing the steps of the methodology.**

Mean reflectivity values per intraretinal layer were normalized to the RPE reflectance and used in the analyses. Mean thickness values per intraretinal layer were obtained by calculating the mean distance between the boundaries comprising each layer. The mean values were calculated per intraretinal layer across the six radial OCT scans. We have previously shown the high repeatability and reproducibility of OCTRIMA measurements [23, 24]. Figure 5 shows a flowchart of the overall methodology. One-way ANOVA was performed followed by Newman-Keuls post-hoc analyses to test for differences between pathological and normal groups. A modified p value of <0.001 was considered statistically significant. Receiver operating characteristic (ROC) curves were constructed to describe the ability of each quantitative parameter to discriminate between eyes of pathological patients and normal healthy eyes. The parameters of interest were the thickness and fractal dimension of each intraretinal layer. Several discriminative diagnostic characteristics of the ROC curve were analyzed. These included the c-statistic (the concordance index, which is the area under the ROC curve used to compare diagnostic power), the sensitivity, specificity, and the positive likelihood ratio (PLR, sensitivity/1 - specificity). The positive likelihood ratio (PLR) combines the sensitivity and specificity at the threshold value by dividing the proportion of true positives by the proportion of false positives. The PLR statistic indicates how likely it is that a case will have an abnormal test compared with a control. The AUROC calculations and statistical analyses were performed using the software package SPSS version 16 (SPSS Inc, Chicago, Illinois).

## Results and discussion

A total of 117 eligible eyes (702 B-scans) were analyzed, which included a total of 74 healthy eyes (34 ± 12 years, 52 female, 22 male), and 43 eyes with mild diabetic retinopathy (MDR, 43 ± 17 years, 21 female, 22 male). The demographic and clinical characteristics of the study population are summarized in Table 1.

**Table 1:** **Descriptive statistics of the study participants**

Characteristic	Controls	MDR
Number of participants	41	29
Number of eyes	74	43
Age (years, mean ± SD)	34 ± 12	43 ± 17
Female, N (% total eyes)	52 (70%)	21 (49%)
Race (% Caucasian)	100	91
Hemoglobin A1c level (%)	-	8.51 ± 1.76
DM duration (years, mean ± SD)	-	22 ± 10
IOP (mmHg, mean ± SD)	14. 5 ± 1.23	15.09 ± 1.56
BCVA	1.0 ± 0.00	0.97 ± 0.06
Total macular thickness (μm ± SD)	324.36 ± 10.27	297.40 ± 21.79

*Abbreviations*: *SD* standard deviation, *BCVA* best corrected visual acuity.

**Figure 6:**
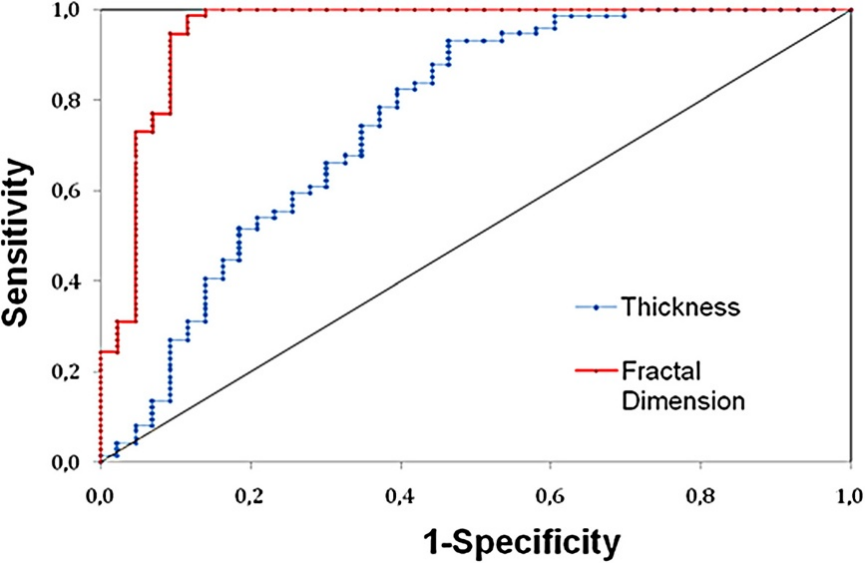
**ROC curve showing the results of the sensitivity and specificity test.** The GCL + IPL complex was used for classifying diabetic retinal tissue with early neural loss based on fractal dimension in OCT images. The AUROC is calculated to be 0.96.

Table 2 shows the thickness and fractal dimension results as well as the outcome of the ROC and statistical analyses. Figure 6. shows a graphic interpretation of the predictive value of the parameters analyzed. The thickness of the GCL + IPL complex, OPL and OS were statistically significantly smaller (8%, 13% & 10%, respectively) in the MDR eyes compared to normal healthy eyes (see Table 2). The thickness in other layers (except in the ONL + IS and RPE) showed a tendency towards thinning without reaching significance as compared to normal healthy eyes. Fractal dimension values were bigger for all the layers (except the GCL + IPL and INL) in MDR eyes compared to normal healthy eyes. When comparing MDR with normal healthy eyes, the highest AUROC values estimated for the fractal dimension were observed for GCL + IPL and INL (see Table 2). Moreover, the highest AUROC values estimated for the thickness measurements were observed for the OPL, GCL + IPL and OS. Particularly, compared to the standard thickness measurement, we found that the fractal dimension of the GCL + IPL complex might be a much better indicator for early DR diagnosis when comparing MDR eyes with control healthy eyes. (see Figure 6). The maximum discrimination value for fractal dimension of 0.96 (standard error =0.025) for the GCL + IPL complex was obtained at a FD ≤ 1.66 (cut off point, asymptotic 95% CI: lower-upper bound = 0.905-1.002). Therefore, there is a 96% probability the diabetic subject will have an abnormal GCL + IPL structure (i.e. disordered structure compared to normal healthy subjects). The ≤ 1.66 threshold coincides with the mean ±2SD for the OCT measurements. At this value, the sensitivity for the GCL + IPL complex is 98% with a specificity of 88%. The positive likelihood ratio for GCL + IPL complex is 15.53, which increase the probability of early retinopathy development about 70%.

**Table 2:** **Distribution statistics of thickness and fractal dimension**

Intraretinal layer	Thickness (microns)					
	Healthy (mean ± SD)	MDR (mean ± SD)	AUROC (mean ± SE)	Asymptotic 95% confidence interval (Lower-upper bound)	Cutoff point	Positive likelihood ratio
RNFL	42.02 ± 2.11	41.38 ± 2.93	0.598 ± 0.059	0.483 - 0.713	41.03	1.51
GCL + IPL	78.30 ± 4.09	71.80 ± 8.22^‡^	0.756 ± 0.05	0.657 - 0.855	75.88	2.90
INL	35.02 ± 1.60	35.05 ± 2.76	0.508 ± 0.061	0.388 - 0.627	34.86	1.33
OPL	41.30 ± 2.49	36.07 ± 3.45^‡^	0.878 ± 0.041	0.797 - 0.958	38.24	6.48
ONL + IS	86.41 ± 5.21	88.39 ± 8.21	0.394 ± 0.055	0.285 - 0.503	86.86	1.07
OS	16.27 ± 3.06	14.40 ± 2.20^‡^	0.688 ± 0.049	0.591 - 0.784	14.59	8.22
RPE	12.71 ± 1.32	12.76 ± 1.09	0.481 ± 0.054	0.375 - 0.588	12.40	3.52
**Intraretinal layer**	**Fractal dimension**					
	**Healthy (mean ± SD)**	**MDR (mean ± SD)**	**AUROC (mean ± SE)**	**Asymptotic 95% confidence interval (Lower-upper bound)**	**Cutoff point**	**Positive likelihood ratio**
RNFL	1.74 ± 0.04	1.78 ± 0.10^‡^	0.393 ± 0.056	0.284 ± 0.503	1.74	1.02
**GCL + IPL**	1.68 ± 0.01	1.58 ± 0.05^**‡**^	**0.953 ± 0.025**	0.905 - 1.002	1.66	**15.53**
INL	1.78 ± 0.01	1.76 ± 0.03^‡^	0.785 ± 0.053	0.680 - 0.890	1.77	3.02
OPL	1.51 ± 0.01	1.56 ± 0.04^‡^	0.111 ± 0.041	0.031 - 0.190	1.52	1.02
ONL + IS	1.78 ± 0.03	1.79 ± 0.04	0.336 ± 0.055	0.228 - 0.444	1.78	2.96
OS	1.70 ± 0.02	1.73 ± 0.04^‡^	0.268 ± 0.047	0.177 - 0.359	1.71	1.00
RPE	1.68 ± 0.01	1.68 ± 0.01	0.433 ± 0.056	0.323 - 0.543	1.68	1.09

Note that mean ± SD, mean ± SE for groups (‡ p < 0.001, SD: standard deviation, SE: standard error), AUROC, cutoff point, confidence interval and positive likelihood ratio values are also included for each variable analyzed. The Fractal Dimension of the GCL+IPL layer had the highest discrimination value (shown in bold).

Our results suggest that the RNFL and GCL + IPL complex, OPL and OS are more susceptible to initial damage when comparing MDR with control healthy eyes. Particularly, the trend observed for the thickness (thinning) of the RNFL and GCL + IPL complex in MDR eyes might be associated with pathological metabolic changes in the retina and may reflect neurodegenerative changes in the diabetic retina. These findings also have possible implications for the early detection of macular damage in diabetes. Interestingly, our results showed for the first time that the thickness of the OPL in MDR eyes was significantly reduced compared with similar measures in normal healthy eyes. Interestingly, a significant decrease in fractal dimension was only observed for the GCL + IPL complex of MDR eyes compared to controls. This result is in agreement with previous reports showing a significant reduction of the fractal dimension during induced apoptosis throughout early apoptotic phases in breast cancer cells [28].

There are limitations to the present study, some of which might be improved in subsequent investigations. First, improved validation of the current methodology demands a larger patient population for analysis. Second, although the TD-OCT technology provides lower image resolution compared to advanced OCT technologies, the six retinal layers were reliably assessed and were the standard when this study was initiated in 2007. However, better results might be expected with advanced OCT imaging technologies mentioned earlier and should be the standard for future studies [23, 24]. Third, although texture measures of the retinal tissue are not standardized measures for detecting significant intraretinal changes, texture-based measures can be obtained from OCT intensity images. Therefore, as reported by previous studies comparing results between TD-OCT and SD-OCT devices, we expect the trends reported here to be replicated by advanced OCT devices [29, 30]. Fourth, FD of the GCL + IPL was not always discriminative. For example, the discrimination power of the FD parameter of the GCL + IPL failed to classify Hispanic subjects. Our study population was overwhelmingly white and Caucasian (see Table 1). Our model using the FD of the GCL + IPL as a risk predictor for early retinopathy showed a good discrimination with high sensitivity and specificity for Caucasain participants. However, a homogenous population does not allow us to probe into the whole scope of the variability in DR risk. Our normal healthy (MDR) subjects were 100 (91)% white/Caucasians (see Table 1). Therefore, our model, which ignored ethnicity and race, could still discriminate well in a population made up entirely of white and Caucasian people, since in these cases ethnicity and/or race is not relevant to their risk relative to one another. In a population of mixed ethnicity, it would discriminate less well the larger the minority group. As a result, calibration and reclassification tasks at specific thresholds reflecting race/ethnicity variability should be in place when using a heteregoneous population in future studies. Fifth, the MDR group was not age-matched to controls in our study. Although aging is known to be associated with loss of complexity in organ structures of the human body due to functional loss, [31, 32] earlier works did not find any correlation between aging and FD of the retinal vasculature [33, 34]. These earlier studies were based on the box-counting method to calulate the FD, which is not the best technique to estimate FD in the presence of segmentation errors due to background noise [35]. However, recent evidence supporting rarefaction of the retinal vasculature has been reported by Zulfaezal et al. [36] . However, the possibility of intraretinal changes as a secondary effect to aging cannot be discarded when comparing MDR with healthy eyes. Therefore, additional work is needed to include more subjects with a broader age range, to fully appreciate the effects on FD from this aging factor using OCT images. Moreover, separating norms by gender is required when designing future studies. Fifth, study parameters were measured cross-sectionally and not longitudinally. Therefore, future studies should investigate whether changes attributed to age could be due to other factors (e.g. sex and race) that may vary between subjects. Six, because patients enrolled in our study were 91-100% Caucasian, results cannot be generalized to other racial populations. Despite these basic limitations, the data presented here reveal that it may be possible to differentiate MDR eyes from normal healthy eyes by analyzing the OCT signal using fractal analysis [37]. More comprehensive studies including investigations on larger subject populations and longitudinal studies using advanced OCT technologies are needed to confirm our preliminary results.

In summary, we have shown that it may be possible to differentiate MDR eyes from normal healthy eyes by analyzing the OCT signal using fractal analysis. The highest AUROC values estimated for the fractal dimension were observed for the GCL + IPL complex in diabetic patients. Particularly, fractal dimension was smaller for this complex in diabetic eyes. A smaller value of this parameter in case of pathologic retinal deformation, or degradation due to apoptosis (cell death) is expected [26]. As cells undergo this apoptosis process, bodies within the cell, like the nucleus or mitochondria, go through structural changes. The use of fractal analysis for classification of diabetes-induced retinal damage in OCT clinical data could potentially provide additional diagnostic information for early detection and progression of DR.

## Conclusions

A potential improvement in the clinical application of OCT to eye diseases is the quantification of the anatomic changes along with the dysfunction of the cellular layers of the neurosensory retina. Our preliminary results suggest that the fractal dimension of the intraretinal layers might provide useful information to differentiate MDR eyes, which are characterized by neurodegeneration at the early stages, from healthy eyes in addition to the structural information. Particularly, the differentiation between normal and abnormal retinal tissue may improve understanding on the sequence of events involved in the visual field defects and provide new insights of the clinical relevance of certain specific morphological features. Further research is warranted to determine how this approach may be used to improve diagnosis of diabetic retinopathy and retinal dysfunction in DR. Specifically, we will have to prove that the fractal dimension is able to discriminate pathological eyes from healthy eyes with higher sensitivity and specificity compared to standard thickness parameters. Thus, the future evaluation of this method using a larger set of data would ultimately lead to a more rational and effective approach to therapy and improved diagnosis. In addition, a more effective classification analysis could be implemented by incorporating other metrics such as depth-dependent attenuation rate and a three-dimensional fractal-based method for 3D OCT data [25, 38–41]. It is important to mention that high resolution is vital for extracting information from OCT images affected by speckle noise. Therefore, the results in our study may be to some extent improved by the utilization of an ultrahigh resolution OCT device [42].

## Abbreviations

OCT: Optical coherence tomography
ROC: Receiver operating characteristic
AUROC: Area under the ROC curve
MDR: Mild diabetic retinopathy
PLR: Positive likelihood ratio
OCTRIMA: OCT retinal image analysis
RNFL: Retinal nerve fiber layer
GCL + IPL: Ganglion cell and inner plexiform layer complex
INL: Inner nuclear layer
OPL: Outer plexiform layer
ONL: Outer nuclear layer
ONL + IS: Inner photoreceptor segment
OS: Outer photoreceptor segment
RPE: Retinal pigment epithelium
FD: Fractal dimension
MS: Multiple sclerosis
SD: Standard deviation
SE: Standard error
2D: Two-dimensional
3D: Three –dimensional.
